# Supplementary material for: Analysis and Research on Starch Content and Its Processing, Structure and Quality of 12 Adzuki Bean Varieties
Source: Foods. 2022 Oct 27;11(21):3381. doi: 10.3390/foods11213381 (PMC9656587; doi:10.3390/foods11213381)
Supplement: Supplementary file 1 [file foods-11-03381-s001.zip › foods-1904655-Supplementary materials.pdf]

Table S1. Properties of starch in different adzuki bean varieties.

Physical and chemical properties	Class I	Class II	Class III
Solubility/%	10.09±0.01	9.64±0.03	9.85±0.02
Swelling power/%	13.72±2.18	14.21±1.67	12.75±0.95
Freezing-thawing stability/%	49.18±3.22	53.63±0.24	49.09±3.28
Amylose/%	13.12±2.37	21.51±9.21	10.29±4.54
Amylopectin/%	86.88±2.37	78.49±9.21	89.71±4.54
AM/AP ratio	15.18±3.22	28.36±15.11	11.77±5.75
Bean paste rate/%	53.35±0.08	47.17±0.03	56.74±0.09
Starch granule size	40.05±4.82	41.38±1.78	38.79±0.47
Hardness/N	137.97±12.52	204.43±43.35	55.08±21.03
Cohesion ratio	0.13±0.04	0.17±0.03	0.11±0.03
Elasticity/mm	1.93±0.58	2.22±0.48	0.88±0.45
Adhesiveness/N	19.10±6.43	36.90±4.01	6.38±2.68
Chewiness/ mJ	40.64±23.07	84.88±5.79	7.00±5.85
Whiteness	129.85±7.69	137.60±3.63	132.50±3.59
RDS/%	57.41±4.79	47.73±1.95	53.81±5.13
SDS/%	25.36±7.82	38.04±5.07	28.84±1.79
RS/%	7.23±4.76	4.23±3.12	7.35±3.96
To/℃	60.95±3.43	70.43±5.06	58.49±4.35
Tp/℃	70.27±0.86	75.49±4.11	75.36±2.91
Tc/℃	83.05±1.06	87.93±6.75	88.21±3.30
ΔH/J/g	3.75±1.01	3.40±1.52	2.71±0.12
Crystallinity/%	76.66±0.63	81.71±5.43	81.78±3.10
